# A Comprehensive Spatial Mapping of Muscle Synergies in Highly Variable Upper-Limb Movements of Healthy Subjects

**DOI:** 10.3389/fphys.2019.01231

**Published:** 2019-09-27

**Authors:** Alessandro Scano, Luca Dardari, Franco Molteni, Hermes Giberti, Lorenzo Molinari Tosatti, Andrea d’Avella

**Affiliations:** ^1^Institute of Intelligent Industrial Technologies and Systems for Advanced Manufacturing, National Research Council of Italy, Milan, Italy; ^2^Department of Mechanical Engineering, Polytechnic University of Milan, Milan, Italy; ^3^Villa Beretta Rehabilitation Center, Valduce Hospital, Costa Masnaga, Italy; ^4^Laboratory of Neuromotor Physiology, IRCCS Fondazione Santa Lucia, Rome, Italy; ^5^Department of Biomedical and Dental Sciences and Morphofunctional Imaging, University of Messina, Messina, Italy

**Keywords:** muscle synergies, centroids, synergies clustering, upper-limb workspace, motor control

## Abstract

**Background:**

Recently, muscle synergy analysis has become a standard methodology for extracting coordination patterns from electromyographic (EMG) signals, and for the evaluation of motor control strategies in many contexts. Most previous studies have characterized upper-limb muscle synergies across a limited set of reaching movements. With the aim of future uses in motor control, rehabilitation and other fields, this study provides a comprehensive characterization of muscle synergies in a large set of upper-limb tasks and also considers inter-individual and environmental variability.

**Methods:**

Sixteen healthy subjects performed upper-limb *hand exploration* movements for a comprehensive mapping of the upper-limb workspace, which was divided into several sectors (Frontal, Right, Left, Horizontal, and Up). EMGs from representative upper-limb muscles and kinematics were recorded to extract muscle synergies and explore the composition, repeatability and similarity of spatial synergies across subjects and movement directions, in a context of high variability of motion.

**Results:**

Even in a context of high variability, a reduced set of muscle synergies may reconstruct the original EMG envelopes. Composition, repeatability and similarity of synergies were found to be shared across subjects and sectors, even if at a lower extent than previously reported.

**Conclusion:**

Extending the results of previous studies, which were performed on a smaller set of conditions, a limited number of muscle synergies underlie the execution of a large variety of upper-limb tasks. However, the considered spatial domain and the variability seem to influence the number and composition of muscle synergies. Such detailed characterization of the modular organization of the muscle patterns for upper-limb control in a large variety of tasks may provide a useful reference for studies on motor control, rehabilitation, industrial applications, and sports.

## Introduction

In recent years, the study of motor control has focused on a human-centered perspective by emphasizing the importance of evaluating muscle or kinematic coordination patterns. Such view focuses on the premise that the Central Nervous System (CNS) relies on a limited number of modules (Bizzi et al., 2008), possibly implemented at neural level (Bizzi and Cheung, 2013), to simplify the production of movement. Consequently, by properly recruiting spatial modules with temporal activation coefficients, the CNS exploits a reduced set of pre-shaped neural pathways, called synergies, to achieve a large variety of motor commands. This view implicitly assumes that, if synergies are encoded at neural level, a unique set should be used across a variety of movements or, at least, task-specific sets should underlie movements requiring similar motor commands. Thus, different movements within a task (e.g., reaching movements in different directions) may be generated by one set of synergies, but different tasks may require different or additional synergies.

The approach based on muscle synergies for the analysis of human motor control has been widely exploited in the literature. Applications of the muscle synergy concept included, among others, investigations of muscles synergies in the upper-limb in physiological conditions (d’Avella et al., 2006, 2008), and the effect of neurological lesions (Cheung et al., 2009, 2012). Synergies have also been applied to locomotion (Ivanenko et al., 2004; Clark et al., 2010; Dominici et al., 2011; Lencioni et al., 2016), and to postural control (Torres-Oviedo and Ting, 2007; Safavynia and Ting, 2013).

While the existence of a modular control architecture has broad acceptance in the literature, many points still need to be clarified for a full comprehension of the framework and its applicability. Addressing some of the open issues, some studies investigated whether a reduced set of synergies might be at the basis of a variety of movement (Valero-Cuevas et al., 2009), whether synergies might be implemented in a sparse fashion (Prevete et al., 2018), and might have a real correlation in the task space, questioning whether the extracted synergies are really able to reconstruct the original movement (Alessandro et al., 2013). Moreover, different algorithms for synergy extraction have been compared (Tresch et al., 2006), and different synergy models –such as time-invariant or spatial synergies and time-varying synergies (d’Avella et al., 2003) have been proposed.

While some open issues are still debated, the impact of the muscle synergies approach has broadened to many fields, as explained in a recent review (Taborri et al., 2018), even outside the motor control field in which the method was conceived. For example, neurological rehabilitation may exploit the potential of the approach for gaining deeper insights concerning motor impairment. Cheung et al. (2009, 2012) suggested that in post-stroke patients, spatial synergies are preserved even if their recruitment timing is altered and that, in severely impaired patients, effects such as fractionation or merging of synergies might be used as biomarkers for assessing motor control alterations. As suggested by other authors (Jarrassé et al., 2014) muscle synergies might be involved in the development and control of rehabilitative exoskeletons, or even assistive robotics. Lastly, muscle synergies might also be employed for the assessment of sports (Taborri et al., 2018).

Following these premises, the screening of the literature clearly highlights the large amount of assessments and applications that may benefit from a comprehensive mapping of muscle synergies for the upper-limb as a reference database. Furthermore, it should be noted that muscle synergy studies are often limited to a reduced number of subjects, or compare specific conditions with others. While in walking, several studies are available to set a reference dataset (such as Clark et al., 2010), possibly also because of the challenges in collecting and analyzing them, large databases of upper-limb synergies are still missing. In fact, the richest available assessment focusing on the upper-limb, explored a variety of reaching movements (point-to-point, reversal, and through via-points) concluding that a set of 4–5 synergies could account for >0.80 of the total variation of the original EMGs, based on 17 muscles recorded on 9 subjects (d’Avella et al., 2006). However, this study analyzed only the frontal and sagittal planes.

Furthermore, when considering the possible exploitation of muscle synergies to real applications, a relevant aspect that has rarely been investigated is the effect of natural high inter-individual variability. In fact, experimental designs are usually confined to stereotyped and constrained scenarios, where subjects have to perform repetitive movements toward a reduced set of targets, and in very controlled postural conditions. These designs consider in detail only target variability, for example with the paradigmatic set of circular targets arranged in 1–2 different planes (such as in d’Avella et al., 2006; Tang et al., 2014; Kieliba et al., 2018 and many others), or in case of some studies, spanning across some gestures (as in the case of rehabilitation in Cheung et al., 2009), while inter-individual/environmental variability, related to natural and ecological variability of motion which may depend on many factors (such as personal attitude, different adopted motor strategies, training, sex, age, target positioning, anatomical mismatching and others), have been usually neglected or strongly constrained. These experimental designs have been employed to analyze specific aspects and thus include controlled conditions. However, a growing literature suggests that variability is a key factor in motor control and motor learning. In fact, it was experimentally shown that action exploration and motor variability facilitate motor learning in humans and that our CNS actively regulate it to improve learning (Wu et al., 2014; He et al., 2016). In the field of muscle synergies, a weakly constrained scenario was considered in the study of multi-directional point-to-point movements (Delis et al., 2018; Hilt et al., 2018). These studies confirmed the hypothesis of modularity in upper-limb movements with limited constrains, suggesting that a reduced number of modules are shared across subjects and underlie point-to-point movements in various directions.

In this scientific context, a comprehensive mapping, investigating the possibility of generalizing results through a large variety of movements and subjects, and considering ecological variability, is still missing. Thus, the aim of this study was to explore the repertoire of muscle synergies of the upper-limb in healthy people in conditions of high spatial variability (i.e., exploration of many different movement directions in an unconstrained set-up). By collecting a comprehensive dataset of EMGs during upper-limb movements in a large workspace, we could: first, provide a reference database of upper-limb muscle synergies to be used for various purposes; second, evaluate the effects of the reduction of dimensionality of the dataset on the accuracy in reconstructing the original dataset of synergies; third, investigate the composition, repeatability and similarity of synergies across subjects and movement directions in conditions of high variability. We also found that, in an experimental design simulating ecological variability, the results of the application of the muscle synergy analysis may show a higher inter-individual variability with respect to previous studies.

## Materials and Methods

**FIGURE 1:**
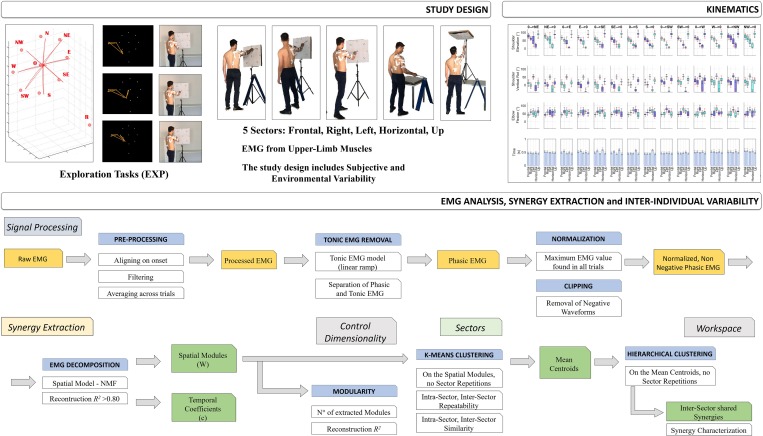
Study Design. The first row of the scheme describes the aim of the work and the study design: to investigate muscle synergies of the upper-limb in a context of high ecological variability. The experimental protocol was performed by 16 subjects (two excluded). Five sectors were investigated: Frontal, Right, Left, Horizontal, and Up, during Hand Exploration Movements (EXP). Tracking data were elaborated on using the Vicon software. Subjects were selected for inclusion and their kinematic and EMG data were filtered, segmented and aligned. Then, kinematics data were extracted (Shoulder Flexion, Shoulder Vertical Rotation, Elbow Flexion, and Movement Time). In the second row, a detailed scheme of EMG data analysis and synergy extraction is portrayed. EMG data were first segmented, aligned, filtered and averaged. Then, EMG data underwent removal of tonic components, to achieve phasic, motion-related waveforms, and were normalized to the maximum EMG on the whole workspace (Workspace Normalization), and lastly clipped to be non-negative. Then, synergies were extracted with the NMF algorithm; the analysis of variability was focused on Variability in Modularity, Variability in Sectors, and Variability in Workspace (see text for details).

An overview of the study design is portrayed in Figure 1.

### Participants

The study took place at the Consiglio Nazionale delle Ricerche (CNR - Italy), UOS Lecco, Human Motion Analysis Laboratory, and under the supervision of the Villa Beretta Rehabilitation Hospital, Costa Masnaga, LC (Italy). The study was reviewed and approved by the CNR Ethical Committee (Rome, Italy). All subjects signed a written informed consent before the experiment, which was conducted in accordance with the Declaration of Helsinki.

Sixteen healthy individuals, neurologically and orthopedically intact, participated to the study. Their data are summarized in Table 1. Due to fatigue and inability to fully complete the protocol, we excluded two subjects from the analysis (subject 15 and subject 16).

**TABLE 1:** Anthropometric data.

**Subject ID**	**Sex**	**Weight**	**Height**	**Age Range**	**Dominant Limb**
1	M	88	180	20–30	R
2	M	80	182	20–30	R
3	M	79	179	30–40	R
4	M	74	180	30–40	R
5	M	73	174	30–40	R
6	F	56	168	20–30	R
7	M	79	183	30–40	L
8	F	51	160	60–70	R
9	M	71	174	70–80	R
10	M	72	179	30–40	R
11	F	56	166	30–40	R
12	F	56	160	20–30	R
13	M	57	171	30–40	R
14	M	80	185	20–30	R
15^∗^	F	65	168	60–70	R
16^∗^	F	60	164	60–70	R

*^∗^Excluded from the analysis.*

### Experimental Set-Up

Subjects stood approximately in the middle of the area tracked by the motion capture system (Vicon 8 TVC system, Oxford, United Kingdom). A support held a target board, with 8 targets indicated by markers placed on a circle of diameter 0.6 m at the cardinal points for movement directions (N, NE, E, SE, S, SW, W, NW), as in previous similar protocols (d’Avella et al., 2006). A 9th marker, labeled 0, was placed at the center of the circle. The distance between each of the peripheral markers and the central marker was of 0.30 m (as in d’Avella et al., 2006). The support was designed so that the set of targets could be freely positioned and oriented in space with respect to the subject. Different positions of the target board were used to map the workspace of the upper-limb. Lastly, a 10th marker (Reference R) indicated the starting position located on the subject’s thigh and was selected by the user in a comfortable position. The requirement for positioning R was not to interfere with movement and being at a lower height than the elbow vertical position.

**FIGURE 2:**
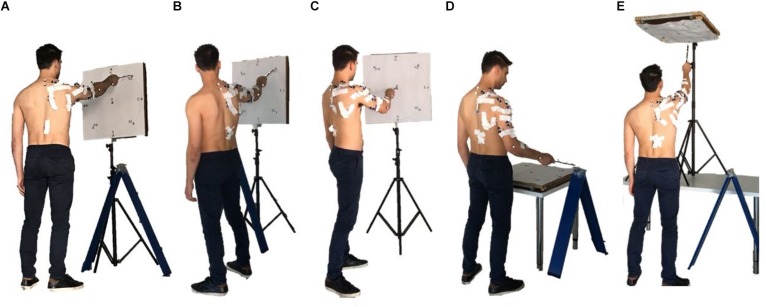
The five set of targets spanning different sectors of the arm workspace considered in the study are portrayed: Frontal **(A)**, Left **(B)**, Right **(C)**, Horizontal **(D)**, and Up **(E)**. Together, they provide a comprehensive mapping of upper-limb workspace.

The acquisition protocol included a comprehensive variety of movement trajectories, considering 5 different positions of the target board with respect to the subject. The targets were oriented Frontal, laterally (Right and Left), upwards (Up), downwards (Horizontal), simulating a mapping oriented toward many of the sectors of the workspace (Figure 2).

The *Hand Exploration Tasks* (EXP) considered in this study are portrayed in Figure 1. EXP movements began with the hand in the center of each target set and consisted in going toward each of the peripheral targets and then coming back to center target. Consequently, each acquisition trial was composed of 16 EXP movements (reaching 8 targets and coming back). The protocol also considered Point-to-Point reaching tasks (PtP), including movements from marker R to each cardinal direction and movements back to the marker R. However, in the present paper, several reasons motivated our choice to present only data from EXP. First, we wanted to compare our results with conditions that have been previously presented in comprehensive similar works (d’Avella et al., 2006); second, due to the richness of the experimental protocol, the methodologies and algorithms for analysis we developed at the present point could not be applied with equal suitability in PtP (see section Data Analysis and Synergies Extraction on data analysis); third, given the aim of the study and the employed design, EXP movements might elicit higher directional variability in respect to PtP and are more suitable for the purpose of the work. After each movement, the subject had to wait for about a second before proceeding to the next target. Furthermore, each subject was asked, for each of the six sets (sectors), to perform ten trials of acquisitions. Subjects were required to move fast, in order to enhance the EMG related to phasic (dynamic) EMG activity. Following this instruction, subjects were expected to complete EXP trials in no more than 0.7 s. However, tolerance in execution time was accepted. To prevent fatigue, after each trial, a pause of 30 s was introduced. The whole protocol had a duration of about 2/3 h per subject. Preliminary assessment of fatigue was performed to verify that subjects were not excessively tired. Results will be not reported since they are beyond the scope of this paper.

During the trials, subjects wore a set of five markers, positioned on D5 and C7 vertebras, acromion (representing shoulder – S), right elbow epicondyle (E), styloid process of the ulna (W). Subjects held a 20-cm long pointer, which was identified by two markers (EE1 and EE2). The recordings were made with the Vicon System (Oxford, United Kingdom). Subjects were instrumented with 14 s-EMG electrodes (Cometa, Italy) positioned according to the SENIAM guidelines (Hermens et al., 2000) on the following muscles: Infraspinatus (IF), Lower Trapezius (LT), Middle Trapezius (MT), Upper Trapezius (UT), Deltoid Anterior (DA), Deltoid Middle (DM), Deltoid Posterior (DP), Pectoralis (PC), Triceps Long Head (TLo), Triceps Lateral Head (TLa), Biceps Long Head (BCl), Biceps Short Head (BCs), Pronator Teres and (PT), Brachioradialis (BR).

### Data Analysis and Synergy Extraction

The first step of the data analysis consisted in pre-processing all the kinematics data with the upper-limb model and target model implemented in the VICON Nexus System. The second step consisted in data elaboration and was performed with Matlab 2018, with custom-developed software.

First of all, kinematic recordings were used to separate movement phases. Each acquisition was thus segmented in 16 movements. The segmentation was achieved by computing the 3D Euclidean distance (3Ed) of the pointing marker from the 0 marker. Then, the velocity profile associated to *3Ed* was computed, and used as signal for detecting movement onsets and offsets.

Furthermore, the kinematics of the upper-limb was computed in intrinsic articular coordinates. Three relevant angles were considered: shoulder flexion, shoulder vertical rotation, and elbow flexion, according to the protocol proposed in a previous study (Scano et al., 2019). Then, in order to compare the data, all the movements were aligned by considering the EMGs in the interval [−0.5; +1.5] seconds with respect to the movement onset. This procedure ensured the capturing the complete EMG waveforms which could begin before movement kinematic onset and finish after having reached the target. The data from 14 sEMG channels were high-pass filtered at 50 Hz (Butterworth filter, 7th order) to remove motion artifacts, rectified, low-pass filtered with a cut-off frequency of 10 Hz (Butterworth filter, 7th order) to extract the EMG envelope. Data from each movement type were intra-subject averaged to characterize a mean pattern, which we labeled “filtered and averaged EMG.” Afterwards, the mean EMG data were further analyzed to extract the phasic component of the EMG, removing the postural (tonic) EMG activity from the original signal (Flanders et al., 1992), following the approach used in previous works (d’Avella et al., 2006). The procedure is graphically shown in detail in the results section. Following this approach, slightly negative EMGs could be obtained in some cases. In order to be able to perform the non-negative matrix factorization (NMF), negative phasic EMG values were set to zero before synergy extraction. This approximation was deemed reasonable since the negative activations were found especially during the backward phases of PtP movements, while in EXP their magnitude was negligible. Lastly, a normalization procedure was performed in order to allow inter-subject comparisons. Since our study design has the distinctive feature of mapping muscle patterns over a variety of movement directions in different portions (or *sectors*) of the upper-limb workspace, we referred the variability captured in the EMG recordings to the whole set of sectors considered at once (whole workspace). Therefore, we decided to normalize the EMG data with respect to a metric considering all the workspace at once. Thus a “Workspace Normalization” of the data was performed on the maximum value achieved for each muscle in the complete dataset (also including PtP movements).

For each subject, the aligned, filtered, averaged, de-ramped, normalized and zero-clipped EMG envelopes were arranged as follows to generate the pooled matrix data to be given as input to the synergy extraction algorithm. For each of the five sectors (Frontal, Right, Left, Up, and Horizontal), all the movements from EXP conditions were grouped together to generate a mapping of each specific sector.

For each of the five sectors, EMG data were pooled together in a pooled matrix data [*EMG(t)*]. Labeling *n*_*s*_ as the number of samples of each movement, *n*_*m*_ as the number of considered muscles (14), and *d*_*r*_ as the number of movements (16 in EXP), data were arranged in [(*n*_*s*_⋅*d*_*r*_) x (*n*_*m*_)] matrices before synergy extraction. Afterwards, spatial synergies were extracted using NMF algorithm (Lee and Seung, 2001). The NMF decomposes the EMG data matrix into the product of two matrices, the first one representing time-invariant synergies (w_*i*_), and the second one representing time-varying activation coefficients for each synergy (c_*i*_), as in equation (1):

(1) $$E\,M\,G\,\left(t\right)=\sum_{i=1}^{N}c_{i}\,\left(t\right)\,w_{i}$$

where *N* is the total number of extracted synergies. Thus, for each decomposition, each spatial synergy was coupled with a set *n* of coefficients (dimensionality *n* = *n*_*s*_⋅*d_*r*_*, i.e., the total number of time samples of all included movements).

The order of the factorization *r* given as input to the NMF algorithm was chosen increasingly from 1 to 14 (the maximum number of muscles that characterizes the maximum dimensionality of the examined control problem). For each *r*, the NMF algorithm was applied 50 times in order to avoid local minima and the repetition accounting for the highest fraction of total variation of the signal explained by the synergy reconstruction was chosen as the representative of order *r*. The number of synergies *N* was then chosen as the minimum *r* explaining at least 80% of the data variation, quantified by a reconstruction *R*^2^ defined as 1 – SSE/SST where SSE is the sum of the squared residuals, SST is the sum of the squared differences with the mean EMG vector (d’Avella et al., 2006). Additional synergies were added only if the total amount of variation explained increased by at least 5% for each further synergy. Solutions of order lower than 3 were not accepted, since the reconstruction of all the directionalities in a plane require a minimum of 3 basic vectors (Russo et al., 2014).

### Analysis of Variability

After the identification of the repertoire of synergies available in the five sectors in each of the two normalization conditions, our aim was to identify if, and at what extent, invariant elements underlie upper-limb movement in conditions of inter-individual variability. In order to do so, we identified three main domains of investigation of the variability, described below. Briefly, *Variability in Kinematics* discusses the variability in intrinsic coordinates across participants, *Variability in Modularity* refers to the variation in the dimensionality of the control space across participants. *Variability in Sectorial Analysis* refers to the variability of the modules in individual sectors, while *Variability in Workspace Analysis* assessed the variability of modules in all the workspace.

#### Variability in Kinematics

At first, we investigated the kinematic variability expressed in articular coordinates, by testing the inter-individual variability within each workspace sector, considering the mean articular angles (shoulder flexion, shoulder vertical rotation, elbow flexion) of each subject for each movement. For each articular angle, we tested the variability both at the beginning and at the end of each movement. Thus, in our tests, sectors were considered separately. In each sector, for each subject and each direction of motion, we stacked the angles for each trial repetition at the beginning and at the end of each movement (0- > N, 0- > NE,…) and tiled them in a vector representing each subject. We tested data for normality using the Kolmogorov–Smirnov test. Then, we tested the inter-individual and inter-directional differences with a 2-way ANOVA with subjects and movement directions as factors. A total of 40 tests = 5 (sectors) × (3 angles + movement time) × 2 configurations (beginning and end of movement) were tested. For all the tests, the significance level was α = 0.05.

#### Variability in Modularity

To investigate modularity of motor control, we first proceeded determining whether different individuals used on average the same number of modules for movement generation in the workspace, tiling together the number of modules for each subject and testing for inter-individual differences. At the same time, we tested if some sectors required more modules than others, tiling together for each sector the number of modules employed by all the subjects, and testing for inter-sector differences. Given the ordinal and not-continuous nature of the variable ‘number of modules,’ these analyses were performed with the non-Parametric Kruskal–Wallis test having subjects (test 1a) and sectors (test 1b) as factors. Following the same procedure, we verified if the amount of reconstruction *R*^2^ was the same depending on subjects (test 2a) and in the various sectors (test 2b) (non-Parametric Kruskal–Wallis test). For all the tests, the significance level was α = 0.05.

#### Variability in Sectorial Analysis

To characterize the synergy repertoire employed in each sector, we performed a clustering analysis. The synergies extracted for each sector across all subjects were used as input to a k-means clustering algorithm (total of 5 cluster analyses, one per sector). In each of the 5 clusterings, the number of clusters was chosen as the minimum that allowed to include in the same cluster no more than one synergy for each subject in the dataset. For the assessment of variability, we introduced two metrics: the *synergy inter-individual repeatability* (from here, simply *repeatability*) and the *degree of similarity within each cluster* (from here, simply *similarity*). Repeatability was defined as the percentage of subjects that share the same synergy on a specific domain (workspace sector). Repeatability provides a measure of the reproducibility across subject of each specific mean synergy (indicating how many subjects over the total share the same synergy). Repeatability was obtained by dividing the number of subjects in each cluster by the overall number of subjects. Instead, similarity was defined as the variability within all the synergies that belong to the same cluster. It provides a measure of the “consistency” of each extracted cluster (indicating how similar are the shared synergies). We computed similarity by averaging the values of all the pairwise dot products between the synergies that compose each cluster.

For each of the five sectors, we first determined the *synergy inter-individual repeatability*, and then we determined whether some sectors had less or more *repeatability* in the usage of modules. We tiled the repeatability of all the centroids of each sector into a vector. After testing for normality with the Kolmogorov–Smirnov test, we performed a one-way ANOVA to test for differences in repeatability across sectors.

We then performed an intra-sector analysis of similarity. We computed the *similarity* of synergies constituting each cluster, as the average of all the pairwise cosine similarity values of the synergies from different subjects within the same cluster. We tiled all the cosine values of all clusters into vectors representing the centroids of each sector, tested for normality with the Kolmogorov–Smirnov test, and performed a one-way ANOVA to test for differences in similarity across clusters, in a way previously proposed in similar studies (d’Avella et al., 2006).

Lastly, we performed an inter-sector analysis of similarity, testing whether there was an effect on similarity due to sectors. We tiled the similarity of all the centroids of each sector into a vector. After testing for normality with the Kolmogorov–Smirnov test, we performed a 1-way ANOVA to test for differences in repeatability across sectors.

#### Variability in Workspace Analysis

Our analysis was concluded with an assessment of the variability of the extracted mean synergies in the entire workspace. The rationale behind this approach was to assess whether different sectors share the same motor modules. If so, it may be possible to further decrease the dimensionality of the dataset of synergies. In order to achieve this result, the set of all the extracted clusters (determined as in section “Variability in Sectorial Analysis”) was used as input to a hierarchical cluster analysis (Matlab “*clustergram*”). We decided to cut the hierarchical tree at the minimum clustering order such that no repetitions of a synergy belonging to the same sector could be found in the same cluster obtained with the hierarchical clustering. In this way, we were able to group similar synergies found within different sectors. With this cutting criterium, we could provide an estimation of the *inter-sectorial repeatability* of clusters, as a summarized result of this work, while their *degree of inter-sectorial similarity* was not controlled due to the choice of the hierarchical tree cutting criterium.

## Results

### Kinematics: Inter-Individual Variability

**FIGURE 3:**
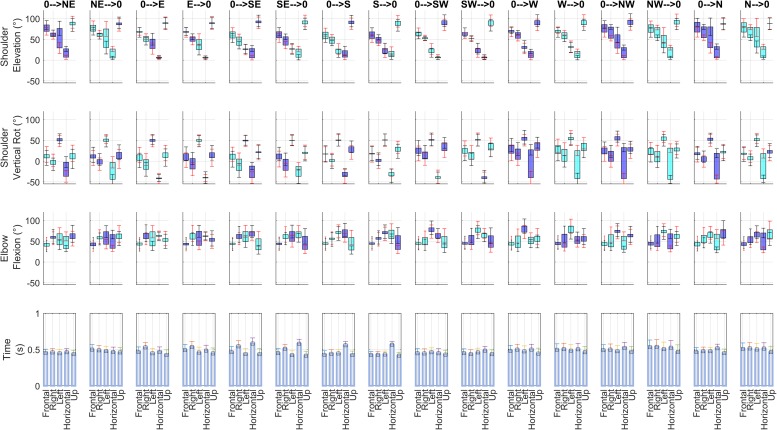
Summary of kinematics. For each of the 16 directions of movement, and for each of the 5 sectors analyzed in this paper, articular angles (Shoulder Elevation, Shoulder Vertical Rotation, Elbow Flexion) and the beginning and at the end of each movement and Movement Time (averaged across the 14 subjects included in the study) are shown. Each articular angle plot (rows 1–3) illustrates the average range of motion for one movement direction (columns) in all five sectors. The following convention are adopted: blue barplots indicate the range of motion when an angle increases from beginning to end of movement, while cyan barplots indicate when an angle decreases. In the graphs of articular angles, black ticks report inter-individual standard deviation for movement end points, while red ticks report inter-individual standard deviation for movement starting points.

The average kinematics of the movements is reported in Figure 3. Interestingly, two-way ANOVA for all considered kinematic variables (3 articular angles at the beginning and at the end of movement, and movement time) revealed that in all sectors there were statistically significant differences among subjects (*p* < 0.001 for all the tests) and among direction of movement (*p* < 0.001 for all the tests; this second result being expected considering that the directions require in general different kinematic patterns). However, these results acquire more interest in the light that the interaction term was significant as well (*p* < 0.001 in all tests), in all the five considered sectors. *Post hoc* tests (not reported in detail) furtherly showed that there were significant interaction effects in many of the combinations of the two factors. These findings allowed us to conclude that in our weakly constrained scenario the adopted motor strategies differ at kinematic level.

### EMGs: Signal Pre-processing

**FIGURE 4:**
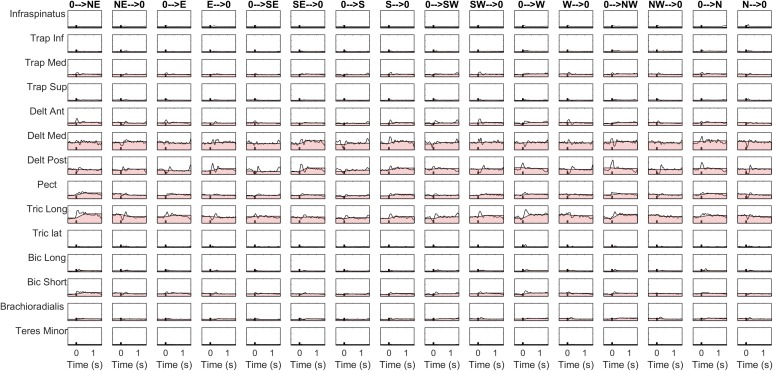
Aligned, filtered and averaged EMG waveforms are reported in light gray for a representative subject and condition. In light red, tonic activity estimated with a linear ramp model is portrayed. The phasic EMG activity is obtained by subtracting the tonic activity from the original envelopes.

**FIGURE 5:**
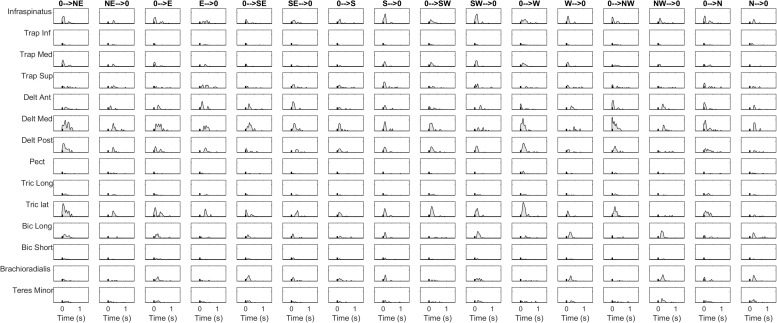
Normalization and zero-clipping procedures have been used to remove the negative phasic components and to apply the standard NMF method for synergy extraction. Black tick indicates movement onset computed with kinematics.

**FIGURE 6:**
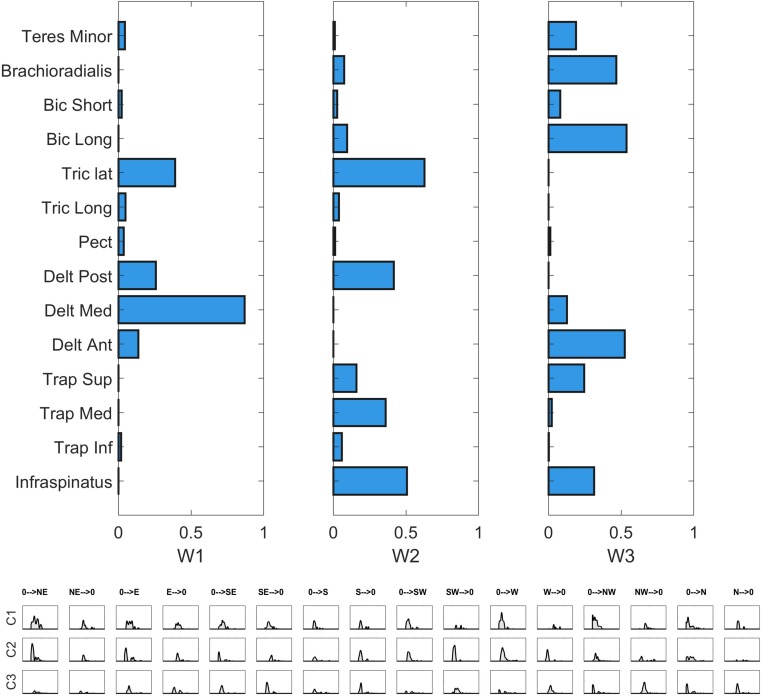
Synergy extraction in a representative condition. In the **upper** panel, the invariant spatial synergies are depicted. In the **middle** panel, the temporal coefficients are portrayed.

**FIGURE 7:**
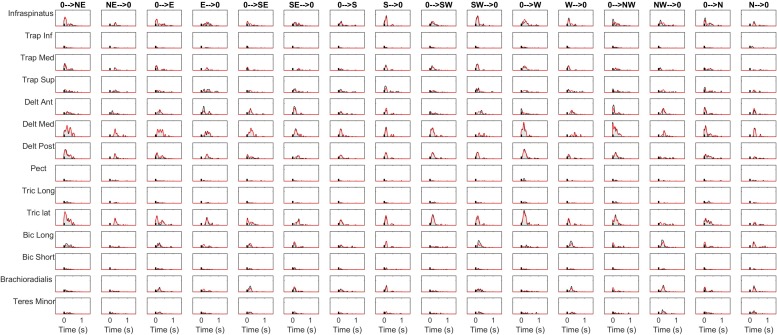
Signal reconstruction in a representative condition. The reconstructed activity is portrayed (red lines) together with the original envelopes (black lines and gray shaded areas).

The details of the EMG pre-processing procedures are shown for a representative subject and condition. Filtered and averaged EMG waveforms, including the contributions from phasic and tonic EMG activity, are portrayed in Figure 4 in light gray, together with the tonic activity estimated according to a linear ramp model, portrayed in light red. In Figure 5, normalization and zero-clipping procedures have been applied in order to remove the negative phasic components and to apply the standard NMF method to extract muscle synergies. In Figure 6, the extracted spatial synergies and the temporal coefficients are illustrated. In Figure 7, the reconstructed EMG envelopes are shown.

### Variability in Modular Analysis

**FIGURE 8:**
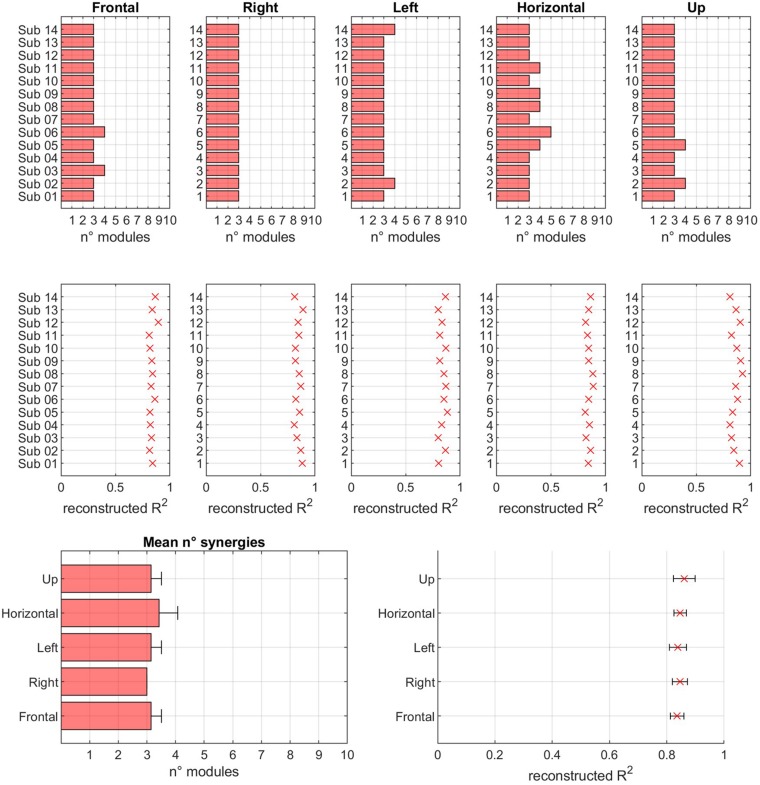
Modularity across sectors. In the first row, for each of sector, the number of modules extracted in EXP movements is reported. In the second row, the reconstruction *R*^2^ is reported. In the third row, the mean values of number of modules and *R*^2^ is reported for each of the considered sectors.

In Figure 8, a summary of the number of extracted modules, for each sector and for both the normalization conditions, and of the corresponding reconstruction *R*^2^ values is reported.

The hypothesis of modular control (i.e., the existence of a reduced set of modules underlying movement) is confirmed. In fact, the number of modules is low with respect to the original dimensionality of the data (14 EMG channels). Thus, the results of this study confirm previous findings that indicate that motor coordination may be achieved by a limited number of modules. In fact, the mean number of modules was 3.17 (Frontal = 3.14, Right = 3.00, Left = 3.14, Horizontal = 3.42, Up = 3.14). The mean *R*^2^ value, corresponding to the mean number of extracted modules reported above, was 0.85 (Frontal = 0.84, Right = 0.85, Left = 0.84, Horizontal = 0.85, Up = 0.86). Further analysis revealed that the number of modules was not significantly different across sectors (*p* = 0.137) and across subjects (*p* = 0.475). At the same time, the *R*^2^ value was not significantly different when considering different sectors (*p* = 0.330), or different subjects (*p* = 0.242). No statistically significant differences were found between subjects, spanning from a minimum average number of modules of about 3 to a maximum of about 3.7. No differences in *R*^2^ were found between subjects (*p* = 0.26).

### Single Sector Analysis

**FIGURE 9:**
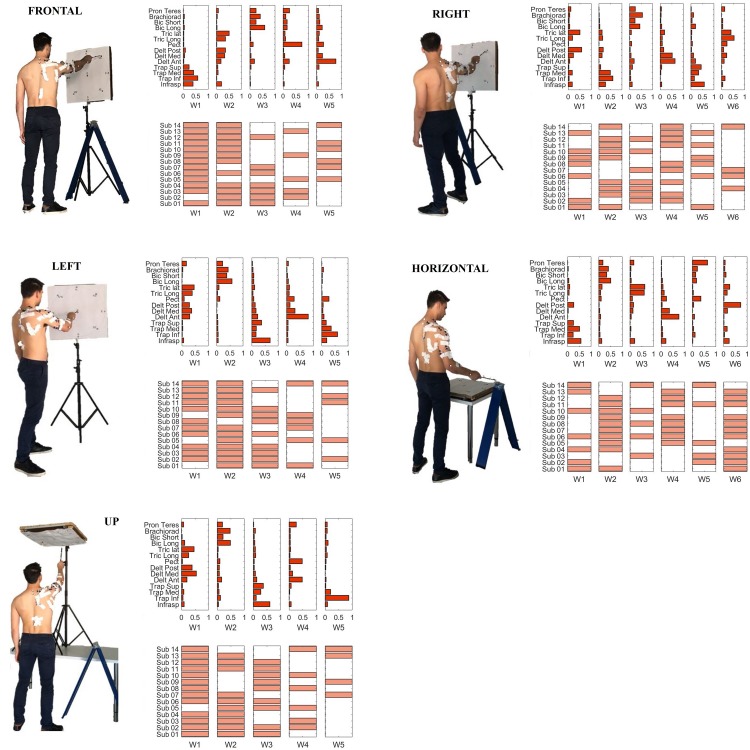
Single sector analysis. Characteristic synergies in the **Frontal, Right, Left**, **Horizontal**, and **Up sectors**. In each sub-plot, in the upper panel, the centroids of the k-means clusters condition are shown. In the lower panel, the inter-individual repeatability matrix indicates which subjects share the same synergies.

We then investigated the composition of the characteristic muscle synergies (centroids of the ten clusters identified by the k-means algorithm). A *repeatability* matrix was computed to identify which subjects employ each synergy in each sector. The results are depicted in Figure 9.

A summary of the main the results for *synergy inter-individual repeatability* and for *the degree of similarity within each cluster* can be found in Table 2. We tested the *synergy inter-individual repeatability* and found that there was no statistically significant difference in the *repeatability* across sectors (*p* = 0.79). The mean of for all the sectors was 57.81% (Frontal = 61.43%, Right = 50.00%, Left = 62.86%, Horizontal = 54.76%, Up = 60.00%). Moreover, we found that there was no sector in which the clusters had significantly higher *degree of similarity* than the others (*p* = 0.67).

**TABLE 2:** Summary of the results for Synergy Inter-individual Repeatability and Degree of Similarity within each cluster identified in each of the sectors.

		**Inter-Individual Repeatability**							

**Repeatability (%)**	**W1**	**W2**	**W3**	**W4**	**W5**	**W6**	**mean**		**Tests**
Frontal	92.86	85.71	50.00	35.71	42.86	//	61.43		No Inter-Sector Difference *p* = 0.79
Right	50.00	64.29	50.00	64.29	42.86	28.57	50.00		
Left	78.57	92.86	64.29	42.86	35.71	//	62.86		
Horizontal	50.00	71.43	35.71	57.14	35.71	78.57	54.76		
Up	92.86	71.43	64.29	42.86	28.57	//	60.00		

		**Degree of similarity within each cluster**							

**Similarity (Normalized)**	**W1**	**W2**	**W3**	**W4**	**W5**	**W6**	**mean**	***p* (Intra-Sector)**	**Tests**

Frontal	0.6222	0.514	0.5188	0.5853	0.7238	//	0.5928	*p* = 0.0062	No Inter-Sector Difference *p* = 0.23
Right	0.6039	0.6724	0.491	0.657	0.557	0.3025	0.5473	*p* = 0.0004	
Left	0.5209	0.6074	0.641	0.8234	0.4357	//	0.6057	*p* < 0.0001	
Horizontal	0.6333	0.4297	0.8052	0.7169	0.4737	0.6242	0.6138	*p* < 0.0001	
Up	0.6733	0.3745	0.4502	0.3537	0.7349	//	0.5173	*p* < 0.0001	

### Variability in the Whole Workspace

**FIGURE 10:**
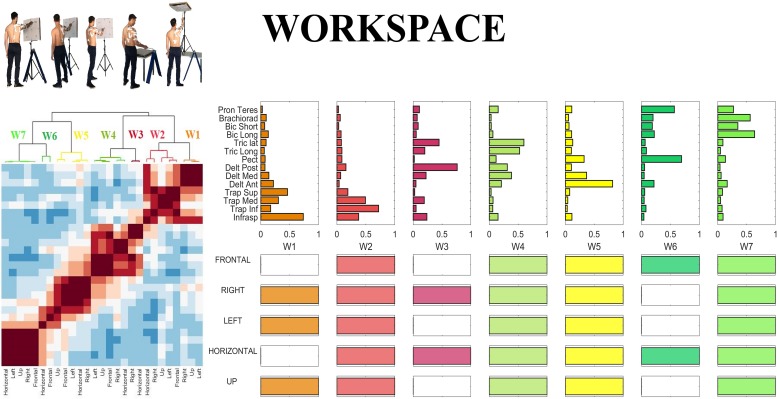
Whole workspace analysis. **(Left)** Clustergram of the similarity matrix of the sectorial centroids. The clustergram highlights the variability between sectors; the dark red square-shaped areas represent the invariant groups of sectorial centroids. **(Right)** Centroids of the hierarchical clustering (whole-workspace centroids) performed on the sectorial centroids and inter-sectorial repeatability matrix, achieved cutting the hierarchical tree at the minimum clustering order needed so that no repetitions of a sectorial centroid belonging to the same sector could be found in the same cluster.

In the last step of our analysis we investigated the *inter-sector repeatability* of the centroids identified in all sectors, as a summary measure portraying which centroids are shared across workspace sectors. In order to do so, we defined the *inter-sector repeatability matrix*, as a workspace homologous of the *inter-individual repeatability matrix* presented in sectorial analysis above. The *inter-sector repeatability matrix* shows which centroids are shared across sectors, and is portrayed in the lower panels of Figure 10. Furthermore, in Figure 10, we reported the Clustergram achieved by pooling together the set of the centroids identified in all five sectors. The hierarchical tree was cut at the minimum order so that there were no repetitions in the *inter-sector repeatability matrix.*

Hereby, we propose a qualitative description of each of the whole-workspace centroids obtained with the clustergram classification.

W1 (shared in Right, Left and Up sectors) is composed of Trapezius (mainly the Superior head) and Infraspinatus. W1 is employed in tasks that involve shoulder external rotation and trunk/limb stabilization. W2 (shared in all sectors) has a similar composition with respect to W1, but with a major contribution of Middle and Inferior Trapezius. As the Infraspinatus activation is lower with respect to W1, this synergy can be employed not only during shoulder external rotation, but especially during arm adduction and limb/trunk stabilization. W3 (shared in Right and Horizontal sectors only) is characterized by the activation of Triceps Lateral head and Posterior Deltoid muscles. W3 is used to achieve posterior and lateral extension of the arm, that are required to explore the set of targets in Horizontal and Right sectors. W4 (shared in all sectors) recruits the three heads of the Deltoid and Triceps Brachii muscles. W4 is used mainly to achieve abduction and external elevation of the limb (due to the activity of the Deltoids), in coupling with Triceps that contributes to the extension of the forearm. W5 (shared in all sectors) recruits majorly the Anterior Deltoid, supported by minor contributions of Medial Deltoid and Pectoralis. W5 is employed in the tasks that require frontal flexion of the upper-limb, especially pointing toward the left hemispace. W6 (employed in Frontal and Horizontal sectors) involves Pronator Teres and Pectoralis. It is used in forearm movements and when the user rotates the arm along the vertical axis in the horizontal plane. W7 (shared in all sectors) is a flexor co-activation pattern, involving the synergistic activity of Biceps Brachii, Pronator Teres and Brachioradialis muscles. It is employed especially when a subject bends the forearm over the arm (elbow flexion).

## Discussion

### Summary of the Main Results

In a loosely constrained scenario, inter-individual differences were found in upper-limb exploration movements at kinematic level. Variability was also found in muscle synergies and was analyzed under various perspectives. First, variability was investigated in the number of extracted modules which had low variation from subject to subject. Accordingly, the mean number of modules was not significantly different from sector to sector. Second, we identified characteristic synergies (cluster centroids) that are available to the group of individuals we tested, for each of the five workspace sectors considered. Variability in muscle synergies was observed in sectors, and it was quantified introducing the concepts of inter-individual repeatability and similarity within each cluster. We found that, on average, repeatability was not significantly affected by the sector, even if, within each sector, some centroids were more repeatable than others. The same conclusion was drawn for similarity: on average, it was not affected by the sectors, even if some centroids had higher similarity than others. Furthermore, we identified which centroids are shared across sectors, and which are specific for some sectors.

### Modular Organization of the Upper-Limb Control

In the recent literature, whether the human CNS exploits a modular organization for the simplification of the motor control problem is debated. In this context, the muscle synergy approach represents the current state of the art for the analysis of the modular organization of the neuro-motor system. The analysis of upper-limb behavior is particularly meaningful, since it is naturally connected to a wide variety of movements, exploiting abundance at both kinematic and muscle level (Latash, 2012), and, consequently, may explore a large variety of conditions.

We found that a number of modules ranging from 3 to 5 reconstructed the original EMG for all subjects in each of the directions. While recent studies suggest that it is arbitrary to neglect a part of the reconstruction *R*^2^, which might be meaningful for movement generation (Barradas et al., 2019), these results are in good accordance with previous studies based on similar reconstruction criteria, which already demonstrated that, in a limited subset of movements of the upper-limb, a 17-muscle model can be reduced to the coordination of five motor modules with time-varying synergies (d’Avella et al., 2006). In the present experiment, starting from a comparable number of EMG channels, a limited number of modules, ranging from 3 to 5, was found for each of the considered mappings. Even if the comparison is done on two different study designs, this result confirms previous findings and expands their applicability to a large variety of upper-limb tasks and movement directions, supporting the concept of modularity also in a wide spatial domain for upper-limb movements. While our analysis relies on a spatial modularity model, evidence for other models such as the time-varying one has been found for similar tasks in previous works. Interestingly, in the full workspace, various inter-sector shared synergies were found to be similar, in terms of muscles contribution, to the ones found by d’Avella et al. (2006) in a smaller workspace with respect to ours and, especially, when the time-varying model was adopted. Referring to the mean synergies “W” found in the work of d’Avella and collaborators (labeled from A to F) and in our study (labeled from 1 to 7), we found the following. WA showed high similarities with W7, except for the contribution of Trapezius Superior, which was not in W7; WB had the same composition (but with a slightly lower contribution from Deltoid Anterior) of W4; WC was identical to W5; WD had high similarity with W2, even if the contributions of Deltoid Middle and Deltoid Posterior are limited. The synergies shared by a limited number of subjects (WE, WF), or sectors (W1, W3, W6) were characterized by a lower similarity. These results suggest that some of the present findings do not depend on the specific model used to describe modules (i.e., spatial synergies or time-varying synergies). Here, considering variability in a large portion of the arm workspace, we found that different sectors of the workspace do not require different numbers of modules and this result was not biased by a different amount of reconstruction *R*^2^. On the same line, in general, different subjects do not require more modules than others. These findings support the hypothesis that control dimensionality is not strongly affected in specific sector of the workspace.

### Analysis of Variability

Variability is intrinsic to human behavior, and this phenomenon can be observed at several levels. Some works rooted human motor variability in reinforcement learning theory, suggesting that motor variability might underlie purposeful exploration of motor space and, when coupled with reinforcement, drive motor learning (Dhawale et al., 2017). Other works state that for any motor task, there are generally a large number of motor-equivalent solutions that can produce functionally equivalent behaviors (Ting et al., 2015; Tommasino et al., 2019), suggesting that human may rely on motor abundance, which is an intrinsic “hardware” source of variability (Latash et al., 2002). Even many aspects of human social communication are related to others’ intention recognition, based on gesture kinematics, and exploit the role of variability (Cavallo et al., 2016). On the contrary, other recent evidence suggests that motor variability may have different effects on learning in redundant tasks: recent studies suggest that, although introducing variability can increase exploration of new solutions, this may come at a cost of decreased stability of the learned solution (Cardis et al., 2017).

In this context, in this work we tried to quantify the amount of variability which is found in a loosely constrained scenario at muscle level. We developed the concept that so far, in the framework of muscle synergies, variability has been mainly connected to target variability, rather than inter-individual and environmental variability. In fact, many study designs are based on very constrained and repeatable scenarios, in which variability is only due to the presence of a limited set of targets. These setups allow, on one side, to draw very specific conclusions on specific questions; on the other side, however, they neglect inter-individual and environmental variabilities, which are respectively subjective and context-dependent sources of variability that are commonly encountered in real-life applications. In fact, the perspective proposed in this study is that real-life applications are not strongly controlled, as people do not follow prescribed endpoint or articular trajectories when reaching for different locations in the workspace (“reach the object” is more prescriptive than “elevate the limb from 0° up to 90°”). In this context, in our experimental protocol, it was expected that the overall variability would increase with respect to previously employed study designs. A first confirmation was found at kinematic level, where in all the experimental conditions, the population adopted significantly different behaviors. Since many aspects may impact on this (anthropometry, sex, age, postural condition, starting point reproducibility, fatigue, etc.), this variability is probably related to inter-subject differences which would be normally found in the real world, and according to many training paradigms, should be mimicked to optimize motor learning or motor-re-learning (Stergiou and Decker, 2011).

In this work, we assess the variability of muscle synergies by quantifying the *inter-individual repeatability* and the *degree of similarity within each cluster*. *Repeatability* provides a high-level measurement of the fraction of subjects sharing a given motor module, while *similarity* describes the solidity of each module (variability of the shared modules). Very interestingly, our results seem to indicate that in general, there is not a “preferred” or “more repeatable” sector of the workspace, which could be expected since some portions of the workspace are less commonly explored than others. In fact, the average repeatability did not differ significantly between sectors, indicating that, on average, there is a comparable inter-individual sharing of modules from sector to sector. The same result was found for the average similarity within each module. However, referring to Figure 9, we can argue that some modules seem to be strongly embedded in people’s repertoire, generalizing through subjects and sectors, while others might be present only in some subjects, or for the fine tuning of specific sectors. This means that the variability of use of muscle synergies is equally spread throughout the workspace, and lower repeatability and similarity are found even in the more frequently used portions of the workspace (such as frontal). However, it should be noted that, when considering a large spatial domain (variability) all together (Workspace condition), the inter-individual repeatability of muscle synergies seems to be significantly decreased with respect to scenarios with less explored workspace presented in previous studies (d’Avella et al., 2006). This result seems to suggest that, when exploring a high-dimensional subset of movements that involve a more variable repertoire, the level of modules (synergies) shared across individuals may tend to decrease. Despite our work involved a remarkable number of workspace sectors, directions and limited constrains, we wonder if a further increase of the natural variability (e.g., different movements, different velocities, different muscle forces, and in general real-life scenarios) would lead to very subject-specific sets of muscle synergies. There are still some analysis choices, such as the criterium for cutting the hierarchical tree, that were considered as the most reasonable, that might have impacted on the definition of the results. Some authors discuss issues related to data analysis and interpretation in more detail (Banks et al., 2017).

Previous works has analyzed similar experimental conditions. In d’Avella et al. (2006), 6 modules underlie several exploration and reversal movements through via-points in two planes. Four modules showed very high repeatability through subjects, supporting a “strong synergy model” (few modules at the basis of a variety of motions, shared across subjects). In the light of our results on a larger spatial domain, this hypothesis seems slightly attenuated, since it is clear that inter-individual repeatability takes place, but at a lower extent for some modules. In fact, we found that repeatability was averagely the same in the five investigated sectors, and on average equal 0.58. It is again clear that employing different data processing, whose choice might be interlinked to the complexity of the study design and the variability of the spatial mapping, may provide different interpretation of the results. In our view, this aspect may alert researchers on the relevance of supporting their findings with the consideration of a wide variety of the movements available to human upper-limb, unless very specific aims suggest otherwise.

Comparable results were found when assessing the similarity of modules. In fact, we found that, on average, the similarity within clusters was the same for all the synergies and that in each portion of the workspace. On the contrary, some synergies are more reproducible (similar) than others when considering the same workspace sector, and thus more solid across subjects.

Other recent studies provided analyses in a scenario comparable to ours. In Hilt et al. (2018), a variety of whole-body point-to-point movements in various directions at a self-selected pace were considered. The authors reported that the spatial modules were not direction-specific but rather functional groups of muscles shared across movements whose weighted recruitment actually codes the task being performed. Furthermore, similarly to our study, they found that the modular control hypothesis is compatible with the observation that different participants could exhibit different motor modules. An additional study focused on intra-individual and inter-individual variability in upper-limb and whole-body workspace during point-to-point movements (Delis et al., 2018). These studies show that a reduced number of synergies may be at the basis of a variety of movement directions, even if the modules might be partially subject-specific, suggesting the relevant role played by variability in a loosely constrained, real-life, experimental scenario.

Lastly, in this study we have not considered another relevant component of variability, which is intra-individual trial-by-trial variability. Recent reviews have shown that trial-to-trial variability in the execution of movements and motor skills is ubiquitous, and widely considered to be the unwanted consequence of a ‘noisy’ nervous system (Dhawale et al., 2017). Furthermore, it has been suggested that motor variability may also be a feature of how sensorimotor systems operate and learn, although the exact mechanisms of how variability affects learning is not well understood (He et al., 2016). Trial-to-trial variability was for instance analyzed in detail in lower-limb muscle synergy analyzing evaluating the effects of concatenation of the EMG data (Oliveira et al., 2014). Considering the aim of the work, which is mainly related to the identification of invariants in a weakly constrained, inter-individual scenario, our choice is reasonable; however, in the light of studies that deepen the role of intra-subject trial-to-trial variability also in whole body and upper-limb applications (Delis et al., 2013), we acknowledge this limitation and will characterize our dataset under this point of view in future works.

### Signal Pre-processing: Normalization and Tonic EMG Removal

While many data processing factors may affect the extraction of muscle synergies, we focus a further discussion of the results on the impact of normalization, which we hypothesized might be significant in our study because of the high variability explored in our dataset. Coherently with the main distinctive feature of our study, which is to provide a mapping of muscle synergies in a wide portion of the workspace, we chose to normalize the EMG envelopes across all the maximum EMG values found in all the trials, because we wanted to refer our results to all the high variability of motions and EMG patterns that we explored throughout the workspace. While this choice is reasonable and coherent, the investigation on our dataset could be further enhanced with Sectorial Normalizations (normalizing EMG data independently from sector to sector) for at least two reasons. First, to allow each sector to have and independent analysis in respect to other sectors; this feature could be relevant when considering future exploitations of our concept, since it would allow experimenters to target only a subset of the proposed analysis depending on the application. Second, since the majority of the proposed study designs so far do not explore workspace variability as much as we did, our results under a workspace normalization perspective are only partially comparable to previous findings. Realizing the relevance of normalization, a recent study (Kieliba et al., 2018) investigated in comprehensive detail the effects of signal preprocessing in an upper-limb experiment, also focusing on the comparison between two different normalization conditions. Kieliba and collaborators found a very high repeatability of the synergies in the two conditions. However, it should be remarked that they used Factor Analysis rather than NMF, employed Maximum Voluntary Contraction rather than the maximum value of muscle envelope in all trials and explored a smaller region of the workspace compared to this study. Among the many pre-processing factors that may affect synergy extraction, we decided to give particular focus on normalization since our condition of Workspace normalization is based on a remarkably larger variety of movement directions than most previous studies. The choice of normalization should be considered when designing experimental paradigms, and evaluate the impact it may have on the results, especially in the framework of muscle synergies. The relevance of normalization (and other processing choices) are discussed also in other studies that introduced a systematic evaluation of the effects of data processing on the extracted muscle synergies in lower-limb analysis (Banks et al., 2017). In the case of lower-limb analysis, different techniques of normalization did not strongly influence the results, but these outcomes cannot be generalized to the case of the upper-limb.

A second relevant aspect of our analysis is the choice of removing tonic EMG components, which are postural components not related to the generation of acceleration and deceleration of the arm. This procedure allowed to separate the phasic components, which are responsible for the motor synergies underlying movement generation. This analysis, introduced in the muscle synergy framework by d’Avella et al. (2006) may have remarkable importance in application scenarios. It has to be acknowledged that the used linear ramp model may introduce approximation in the estimation of the tonic EMG component and more refined models should be developed, also in the light of capturing phasic negative waveforms that are neglected in this study. However, considering clinical test of post-stroke patients as a possible exploitation scenario for this dataset, in which the reference data presented in this study can be compared to post-stroke patients’ motor performance or to robot-assisted movement, this procedure allows, for example, to remove abnormal patterns in static posture and to extract synergies only from movement-related EMG, reducing the chance of misinterpretation of the extracted muscle synergies.

Lastly, the tonic EMG removal may have an impact on the composition, as well as number, of the synergies extracted. This is particularly relevant in applications where the focus is exactly to determine the effect on muscle synergies in weight support applications. This is the case of a study where the effect of a device for the support of the upper-limb weight was investigated (Coscia et al., 2014); in that study, the authors extracted muscle synergies without separation of the phasic and tonic components. They found that a set of 8 spatial synergies underlie a set of reaching movements in the frontal plane. Spatial synergies were not modified when using the device. On the contrary, the temporal coefficients where scaled proportionally to the amount of weight support provided. In this condition, it is likely that some synergies may capture the tonic components and thus the extracted repertoire is a linear summation of phasic and tonic synergies. It is thus expected that, in general, the number of extracted motor modules may increase without tonic EMG removal. For future applications, the separation of the EMG components might be considered to evaluate separately the effect of a device on phasic components (related to motion) and tonic components (related to static weight support). A desirable weight-support device should not alter phasic synergies with respect to free motion, reducing instead the burden associated to tonic synergies. Such a result may be achieved with an even more accurate tonic EMG removal algorithm.

### Application of the Study to a Variety of Scenarios

As suggested in a recent review (Taborri et al., 2018), the muscle synergy approach is a flexible and versatile tool that can be employed in several scientific fields. According to our view, the results and the methodology adopted in this study are potentially applicable to various scenarios described below.

Muscle synergies have been frequently employed in rehabilitation scenarios. Studies from Cheung et al. (2009, 2012) have studied how upper-limb spatial muscle synergies are organized in patients with different level of impairment, in a repertoire of different upper-limb motion. While high functioning patients had physiological-like synergies, low functioning patients suffered of merging and fractionation issues, and the number of available modules was modified accordingly. Other studies have investigated a variety of upper-limb reaching movements and suggested that stroke induces abnormal coordination of muscle activation in severely impaired hemiparetic individuals by altering the structure of muscle synergies (Roh et al., 2013) or that alterations in the shoulder muscle synergies in stroke appear in an impairment level-dependent manner (Roh et al., 2015). Other studies provided on overview of post-stroke patient grouping in one-directional reaching movements (Scano et al., 2017). The examples reported above show how muscle synergies could be used as biomarkers of motor disability. However, in the light of this study, future studies may consider to include a higher movement variability and, in this perspective, the proposed results have a twofold application to rehabilitation. First, this methodology, properly tuned depending on the application, could be used to design evaluation protocols or training paradigms to be performed in free movements, or robot assisted ones. In this view, we suggest the importance of assessing different methodological approaches (including synergy extraction algorithms and EMG normalization), allowing experimenters to consider also subsets of the proposed sectors and movement directions (it is probably unrealistic to use the proposed paradigm in standard clinical use). Testing the motor capability of a neurological patient could be done with specific tests that replicate the proposed methodology to verify the dimensionality and the composition of motor modules available to people. Furthermore, this work may suggest, when possible, to expand the domain of classical paradigms of rehabilitation (Frontal or Horizontal plane), and, compatibly with patients’ capabilities, explore a large variety of movements and sectors.

Moreover, variability has been often considered in the literature dealing with motor control assisted by robotic devices for rehabilitation. Robots for rehabilitation are in fact designed to allow patients to explore the effort-error relationship needed for motor re-learning (Morasso et al., 2009). In the context of promoting rehabilitation, designing robots and assessing their effects in a context of motion variability may enhance their efficacy, as well as matching the assessment of variability with muscle synergies. Following these premises, the evaluation of robot-assisted training seems a natural match with the muscle synergies framework, and in fact preliminary works have exploited this concept, assessing human-robot interaction with muscle synergies (Tropea et al., 2013; Coscia et al., 2014; Lunardini et al., 2016; Pirondini et al., 2016; Chiavenna et al., 2018; di Luzio et al., 2018; Scano et al., 2018). The database here presented, properly expanded and matched with patients, could be a milestone reference point for designing training paradigms or provide in depth evaluations, extending the results found in previous works (d’Avella et al., 2006).

Lastly, in the human-centered perspective promoted in recent industrial applications, bioengineering approaches might be helpful in evaluating the use of a device and understanding its level of support, user-perceived transparency, discomfort and ergonomic features. For example, the Up sector proposed in this study might require a set of the primitives employed when dealing with screwing or overhead tasks, and may be used as a metric for the evaluation of the effects of a device. Future applications of the proposed concept may also include sports, considering that synergies have been applied to cycling, rowing, swimming, ice hockey and fitness (for a detailed review, see the work of Taborri et al., 2018), and could be potentially be employed for a variety of assessment in the sports field. We believe that future applications will also include these topics and field of application.

### Limitations and Future Works

Given the ambitious objectives proposed by this study, a detailed analysis of its limitations and range of applicability is hereby provided. At first, it should be remarked that the use of 14 EMG channels, while being about in line with the standard used in reference articles in the literature (d’Avella et al., 2006), is still limiting considering the huge amount of motor units in the human body. Several authors (Steele et al., 2013) warn about the use of a too small number of channels, suggesting that some modules might be missed. Furthermore, while the synergy approach has been showed to be compatible with experimental findings, its relevance to the effector space must be demonstrated (Alessandro et al., 2013). In this study, as well as in the majority of muscle synergy works, it is not demonstrated that the set of extracted synergy is able to define the same motor output as the original muscle patterns.

Secondly, while being comprehensive and inclusive of variability, the proposed mapping is still limited by the adoption of laboratory movements not yet integrated in real-life scenarios and realistic tasks. Boundary conditions related to interaction with objects or force application were not considered in this study. Furthermore, movements were performed at self-selected, natural speed. A systematic evaluation of the effect of velocity was not investigated.

Moreover, the presented analysis neglects the negative phasic EMG waveforms. While this approximation is reasonable in the presented dataset (EXP movements), future exploitation of the dataset including PtP movements will consider algorithms for extracting synergies from negative waveforms, or the offsetting the data for the removal of negative activations. While the range of applications may vary and include several different scientific fields, it is likely that, depending on the applications, the findings of this study might be furtherly refined. For example, the application of this database to neurological patients’ performances might benefit from fine-tuned recordings for the matching of the reference database to the peculiar features of motor impairment (e.g., reduced range of motion, jerky movements, lack of repeatability), which were not investigated in this study. Future works will investigate the potential of the human-centered, muscle synergy approach for the detailed assessment of specific experimental conditions, related to rehabilitation, medicine, industry, or sports, as well as including a relevant part of the dataset related to point to point movement.

## Conclusion

In this paper, muscle synergies were extracted from the EMG recordings during the performance of upper-limb exploration movements in a large portion of the upper-limb workspace. The variability of the extracted synergies was investigated to evaluate the repertoire available to healthy people.

We found that a limited number of motor modules, modulated by activation signals, underlies the execution of a large variety of upper-limb exploration movements. However, spatial synergies were not always repeatable across subjects and similar within coherent groups, as an effect of variability. In general, considering a wide repertoire of movements, as well as reducing the imposed constrains, leads to the identification of a more flexible modular architecture with respect to the ones identified in previous studies.

## Data Availability Statement

The datasets generated for this study are available on request to the corresponding author.

## Ethics Statement

The studies involving human participants were reviewed and approved by the CNR Ethical Committee (Rome, Italy). The patients/participants provided their written informed consent to participate in this study. Written informed consent was obtained from the individual(s) for the publication of any potentially identifiable images or data included in this article.

## Author Contributions

AS, AD’A, and LD: conceptualization, formal analysis, data curation, and software. AS and LM: funding acquisition. AS, AD’A, LD, and FM: investigation and methodology. AS, LM, and AD’A: project administration. AS, LM, LD, HG, and AD’A: resources. AD’A, AS, LM, FM, and HG: supervision. AS, AD’A, LD, LM, FM, and HG: validation, visualization, and writing – original draft, review, and editing.

## Conflict of Interest

The authors declare that the research was conducted in the absence of any commercial or financial relationships that could be construed as a potential conflict of interest.
